# Identification of CircRNA-Related ceRNA Networks in the Longissimus Dorsi of Yaks at Different Developmental Stages

**DOI:** 10.3390/ani16101497

**Published:** 2026-05-13

**Authors:** Binyan Yu, Xiaoming Ma, Xiaoyun Wu, Min Chu, Xian Guo, Yongfu La, Chunnian Liang, Ping Yan

**Affiliations:** 1 Animal Science Department, Lanzhou Institute of Husbandry and Pharmaceutical Sciences, Chinese Academy of Agricultural Sciences, Lanzhou 730070, China; 17899318984@163.com (B.Y.); maxiaoming@caas.cn (X.M.); wuxiaoyun@caas.cn (X.W.); chumin@caas.cn (M.C.); guoxian@caas.cn (X.G.); layongfu@caas.cn (Y.L.); 2 Key Laboratory for Yak Genetics, Breeding, and Reproduction Engineering of Gansu Province, Gansu Provincial Key Laboratory of Yak Breeding Engineering, Lanzhou Institute of Animal Husbandry and Veterinary Medicine, Chinese Academy of Agricultural Sciences, Lanzhou 730070, China

**Keywords:** RNA sequencing, CircRNA, Datong yak, longissimus dorsi, skeletal muscle development

## Abstract

We investigated the expression profiles and functions of CircRNAs in the longissimus dorsi muscle of Datong yaks at different developmental stages, with the aim of clarifying their regulatory roles in skeletal muscle development. Samples of longissimus dorsi muscle were collected from Datong yaks at three developmental stages: 90-day-old fetuses, 6-month-old juveniles, and 3-year-old adults. High-throughput RNA sequencing was performed to identify CircRNAs. Differential expression analysis, along with GO and KEGG enrichment analyses, was conducted. A competing endogenous RNA (ceRNA) regulatory network was subsequently constructed to screen for core CircRNAs. A total of 17,027 CircRNAs were identified, with 6821 being differentially expressed. These differentially expressed CircRNAs showed significant enrichment in skeletal muscle development-related functions, such as sarcomere and calcium ion homeostasis, and were involved in key pathways, including the FoxO (Forkhead box O) and calcium signaling pathways. Construction of the ceRNA network revealed 20 core CircRNAs (e.g., CircRNA_10402 and CircRNA_15445), which may modulate the expression of 84 mRNAs by competing for binding with 42 miRNAs. This study preliminarily reveals the dynamic regulatory network of CircRNAs during skeletal muscle development in Datong yaks, providing new theoretical insights for understanding the molecular mechanisms underlying yak muscle development and for molecular breeding.

## Simple Summary

Yak meat is a vital food source for communities living on the Qinghai–Tibet Plateau. Improving the quality and yield of yak meat requires a deeper understanding of how yak muscles grow and develop. In this study, we investigated small circular RNA molecules in the longissimus dorsi muscle of female yaks at three life stages: 90-day-old fetuses, 6-month-old calves, and 3-year-old adults. We identified thousands of circular RNAs, whose levels change significantly as yaks grow. Some of these circular RNAs appear to act as master regulators, controlling the activity of other genes involved in muscle formation, structure, and function. By mapping how these molecules interact, we identified 20 key circular RNAs that likely play important roles in muscle development. This work provides new insights into the biological processes underlying yak muscle growth and offers valuable clues for future breeding efforts aimed at improving yak meat quality.

## 1. Introduction

The Qinghai–Tibet Plateau, hailed as the ‘Roof of the World’, presents a formidable challenge to life due to its extreme cold, low oxygen, and intense ultraviolet radiation. The yak, a precious domesticated animal bred in this region, offers herders vital resources including meat, milk, and hides, while also serving as a crucial draught animal [1]. China accounts for ~95% of the global yak population (over 16 million heads), with Qinghai Province alone housing more than 5 million heads. In 2023, China’s yak meat output exceeded 650,000 metric tons (mt), and the total output value of the yak industry reached approximately 499 billion RMB. As the primary animal protein source for Tibetan herders, yak meat yield and quality directly determine the economic benefits of the plateau animal husbandry industry and the livelihood security of pastoral households [2,3]. Therefore, thoroughly exploring yak’s meat production potential and systematically enhancing its meat performance have become the primary tasks of current genetic breeding research.

The meat production performance of livestock and poultry fundamentally depends on the efficiency of skeletal muscle growth and development [4]. Numerous research has established that yak meat possesses distinctive quality attributes, including a high protein content (approximately 20%), low intramuscular fat (approximately 3%), and enriched levels of essential amino acids and polyunsaturated fatty acids, particularly eicosapentaenoic acid (EPA) and docosahexaenoic acid (DHA), compared to conventional beef. Furthermore, yak meat from grazing systems on the Qinghai–Tibetan Plateau exhibits a favorable n-6/n-3 polyunsaturated fatty acid ratio, meeting nutritional recommendations for healthy food [5]. Meat quality characteristics including tenderness, water-holding capacity, and shear force vary significantly with age, sex, feeding regime, and muscle location [6]. Skeletal muscle development constitutes a multi-stage, highly complex and precisely regulated biological process, commencing within embryonic mesodermal somites. It involves the proliferation, differentiation and fusion of myoblasts, ultimately forming multinucleated muscle fibers [7]. The process persists across multiple developmental stages, including fetal, juvenile, adolescent, and adult phases, and is characterized by progressive increases in both muscle fiber number and cross-sectional size, along with a concomitant shift in metabolic phenotype [4]. This sequence of events is governed by multi-tiered molecular networks, traditionally dominated by a transcriptional cascade centered on myogenic regulators such as MyoD, Myogenin, and Myf5, alongside associated signaling pathways (e.g., Wnt, TGF-β) [8]. Recent studies indicate that epigenetic regulation, particularly the involvement of non-coding RNAs, significantly enhances the complexity and precision of this network [9]. Therefore, further studies are necessary to explore the post-transcriptional regulatory functions of non-coding RNA and to clarify the mechanisms governing skeletal muscle development.

Among the diverse classes of non-coding RNA, CircRNAs have recently gained considerable attention as a research hotspot, primarily due to their covalently closed circular configuration, enhanced molecular stability, and distinct tissue- and developmental stage-specific expression profiles. CircRNAs are resistant to degradation by RNA endonucleases, possess a relatively long half-life [10], and exhibit highly specific expression patterns, suggesting their critical role in specific biological processes [11]. In skeletal muscle development, CircRNAs primarily exert their effects through the ceRNA mechanism: acting as ‘molecular sponges’ to sequester miRNAs, thus releasing their inhibitory effects on target mRNAs and forming intricate regulatory networks [12]. Multiple studies on livestock muscle development have validated this mechanism. For instance, Li et al. discovered stage- and breed-specific expression of CircRNAs in porcine skeletal muscle, with circFGFR4 regulating the Wnt pathway by binding miR-107, thereby influencing myoblast proliferation [13]. Wei et al. reported that bovine circFUT10 functions as a sponge for let-7, promoting myoblast proliferation while inhibiting differentiation [14]. These studies confirm the significance of CircRNAs and their ceRNA networks in livestock muscle development. Although progress has been made in species such as pigs and cattle, the role of CircRNAs in yak muscle development remains at an exploratory stage. Existing studies have laid the groundwork for subsequent investigations: Wang and Sun et al. utilized high-throughput sequencing to reveal CircRNA expression specificity in yak longissimus dorsi muscle across developmental stages and in comparison with domestic cattle [15]. Nevertheless, the mechanisms by which these CircRNAs dynamically regulate gene networks through ceRNA interactions to drive the transition from rapid juvenile growth to adult muscular maturity in yak remain unclear.

This study selected the dorsal longissimus muscle tissue from 3-year-old, 6-month-old, and 90-day-old fetal yaks. Utilizing high-throughput sequencing technology, we systematically identified differentially expressed CircRNAs across these three developmental stages and constructed corresponding ceRNA regulatory networks. Through bioinformatics analysis, we further explored the biological processes and signaling pathways regulated by this network, identifying core ceRNA regulatory axes that play pivotal roles in muscle developmental transitions. This study establishes a theoretical basis to clarify the molecular mechanisms governing yak muscle development and to support genetic improvement strategies.

## 2. Materials and Methods

### 2.1. Ethics Statement

All experimental procedures involving yaks were performed in strict accordance with the national regulations and ethical guidelines for animal care of the People’s Republic of China. The study protocol was reviewed and formally approved by the Animal Management and Ethics Review Committee of the Lanzhou Institute of Animal Husbandry and Veterinary Medicine, Chinese Academy of Agricultural Sciences (Approval No. 2019-002), ensuring compliance with institutional and national ethical standards.

### 2.2. Sample and Animal Tissue Processing

This study’s experimental yaks were sourced from the Ashidan Mountain area in Qinghai Province. These yaks were raised under standardized high-altitude pastoral conditions, grazing freely on native alpine forage and drinking natural spring water. Their diet was rich in fiber and contained high levels of minerals. All yaks were managed under the same natural environmental conditions, received no artificial feed supplements, and were in good health prior to sampling. From this region, nine healthy female yaks were selected from the same farm, all maintained under the aforementioned husbandry conditions, and divided into three age groups: 6-month-old group (Group M), 3-year-old group (Group A), and 90-day-old fetal group (Group E). Required samples (longissimus dorsi muscle) were collected post-slaughter, cut into 0.5 cm^3^ tissue blocks, treated with RNAlater (Qiagen, Hilden, Germany), and incubated overnight at 4 °C. Samples were subsequently transferred to cryogenic tubes and rapidly frozen in liquid nitrogen before final storage at −80 °C for total RNA extraction, transcriptomic sequencing, and qPCR analysis.

### 2.3. Transcriptome Sequencing and Assembly

Longissimus dorsi muscle tissue was used to extract total RNA with TRIzol reagent (Invitrogen, Carlsbad, CA, USA), followed by quality assessment. RNA integrity was checked via 1% agarose gel electrophoresis. The Agilent 2100 system (Agilent, Santa Clara, CA, USA), along with the RIN value and 28S/18S ratio analysis, was employed to determine the total RNA concentration and purity, thereby ruling out potential contamination. To enrich for circular RNAs, 3 U of RNase R (Epicentre, Madison, WI, USA) was used to digest linear RNA, according to the manufacturer’s recommendations. Subsequently, library preparation was performed using the TruSeq^®^ Total RNA Library Preparation Kit (Illumina, San Diego, CA, USA). Following DNase I (Fermentas, Vilnius, Lithuania) digestion to remove genomic DNA, cDNA was synthesized from 2 μg of total RNA template on the Illumina HiSeq™ 2500 system (Illumina, San Diego, CA, USA). Following quantitative analysis of the libraries using an Agilent 2100 Bioanalyzer (Agilent, Santa Clara, CA, USA), specific-strand libraries were sequenced on the Illumina HiSeq™ 2500 instrument (Illumina, San Diego, CA, USA), generating 150 bp paired-end reads. All library preparation and sequencing were performed on the Illumina platform by OE Biotech Co. (Shanghai, China).

For the raw sequencing data obtained, quality control was performed using the FASTQC tool. Due to a significant decline in read quality observed at the 3′ end, sliding window trimming was conducted using Trimmomatic software (version 0.39), with trimming executed when the average base quality fell below a Phred score of 10% (equivalent to 90% accuracy). The high-quality trimmed reads were re-evaluated using FASTQC (v0.11.9) prior to alignment against the yak reference genome using HISAT2 (v2.1.0). Subsequently, StringTie (v1.3.1) was used to assemble transcripts from the aligned reads for each sample. The resulting transcripts were then integrated and annotated using the Cuffcompare program within the Cufflinks package (v2.2.1).

### 2.4. Identification and Characteristic Analysis of CircRNA

Following acquisition of extensive paired-end sequencing data, we employed Trimmomatic software (version 0.39) [16] to construct a full transcriptome library. High-quality data were obtained by removing adapter sequences and low-quality base reads. Subsequently, Hisat2 (v2.1.0) [17] was employed to align the cleaned sequencing reads against the reference genome BosGru_v2.0, thereby obtaining gene positions and sample-specific sequence information. The RseQC analysis suite was then utilized to statistically analyze the proportion of alignment types and evaluate the results. Following the generation of BAM files, StringTie software (v1.3.1) [18] was employed to assemble and align gene reads, fusing transcripts from each sample into complete transcripts. Leveraging the structural characteristics and splicing sequence features of circular RNAs (CircRNAs), CIRI software (version 2.0.6) was utilized to predict, classify, and annotate CircRNAs, followed by statistical analyses of their chromosomal distribution and length [19]. The analysis was performed using default parameters, with a minimum back-splicing junction support threshold of ≥2 unique reads. The reference genome employed was BosGru_v2.0.

### 2.5. Screening of DE CircRNAs and GO/KEGG Enrichment Analysis of Source Genes

We conducted differential expression analysis on CircRNAs by employing the edgeR package (v3.12.1) [20]. By comparing differences among the 6-month-old (M group), 3-year-old (A group), and 90-day fetal age (E group) cohorts, the entire analytical workflow comprised three principal steps: (1) standardization of circular RNA read counts; (2) calculation of *p*-values for differential expression hypothesis testing based on generalized linear models; (3) applying multiple hypothesis correction with an FDR threshold of 0.05, CircRNAs showing FDR < 0.05 and |log2FC| ≥ 1 were identified as significantly differentially expressed in accordance with the differential analysis results [19].Further analysis of the host genes for these CircRNA was conducted using both the Gene Ontology (GO, https://www.geneontology.org/ accessed on 3 July 2025) and the Kyoto Encyclopedia of Genes and Genomes (KEGG, http://www.genome.jp/kegg/ accessed on 5 July 2025).

### 2.6. Validation of Differentially Expressed CircRNAs

To validate the accuracy of the RNA sequencing data, six differentially expressed CircRNAs were randomly selected and quantified using real-time quantitative PCR (qRT-PCR). β-actin was employed as the internal reference gene for normalization. All primers were custom-designed and synthesized by Qingke Biotechnology Co., Ltd. (Beijing, China) and are listed in Table S1. PCR amplification was performed using cDNA from the longissimus dorsi muscle tissue of the Datong yak as a template, with amplification products subsequently analysed via Sanger sequencing. We aligned the amplified sequences with circular RNA sequences obtained from RNA sequencing using MEGA7.0 software. Next, qRT-PCR was conducted using SYBR Premix Ex Taq II (Takara Bio, Kusatsu, Japan) on a Roche LightCycler^®^ 96 real-time PCR system. The thermal cycling protocol comprised an initial denaturation step at 95 °C for 30 s, followed by 40 amplification cycles of denaturation at 95 °C for 5 s and annealing/extension at 60 °C for 30 s, according to standard amplification conditions. β-actin served as the internal control, and the relative expression levels of circular RNAs were calculated using the 2^−ΔΔ*C*t^ method.

### 2.7. Building a CircRNA–miRNA–mRNA Regulatory Network

Prediction of circular RNA targeting miRNAs employed miRanda software (v3.3a) and TargetScan (v7.0) [21]. Circular RNA–miRNA–mRNA interactions were predicted using the miRTarBase database (v6.1) [22].

### 2.8. Statistical Analysis

For this study, we carried out statistical analysis with SPSS 25.00 software. The experimental results are shown as the mean ± SE (standard error of the mean). Data obtained from RT-PCR were subjected to relative quantitative analysis via the 2^−ΔΔ*C*t^ method. Independent samples *t*-tests or one-way ANOVA were applied for intergroup comparison of differences. Statistical significance was established at *p* < 0.05, and high statistical significance at *p* < 0.01.

## 3. Results

### 3.1. Overview of Sequencing Data

To investigate the expression patterns of CircRNA in the longissimus dorsi muscle of the Datong yak during development, we performed high-throughput sequencing on longissimus dorsi muscle tissue samples from fetuses at three distinct developmental stages: 90 days (E1, E2, E3), six-month-old calves (M1, M2, M3), and three-year-old adults (A1, A2, A3). cDNA libraries were constructed for these three developmental stages. Following sequencing on the Illumina HiSeq 2500 platform, a total of 941.08 million raw reads were obtained. After filtering out low-quality reads and adapters, we obtained 910.59 million clean reads or about 96.76% of the original raw reads in the library. The Q30 value exceeded 96.87% for all samples, validating the reliability of the sequencing data and providing a robust foundation for subsequent analyses. Alignment of the valid data against the reference genome revealed that over 93.10% of the sequenced data achieved precise matching with the yak reference genome, representing a significant improvement in alignment rates. Among these, 9.10–14.57% of valid data exhibited multiple alignment sites, while 77.44–85.01% possessed only a single alignment site. The latter group may serve as reference samples for subsequent bioinformatics analyses (Table 1).

### 3.2. Identified CircRNAs

**Figure 1:**
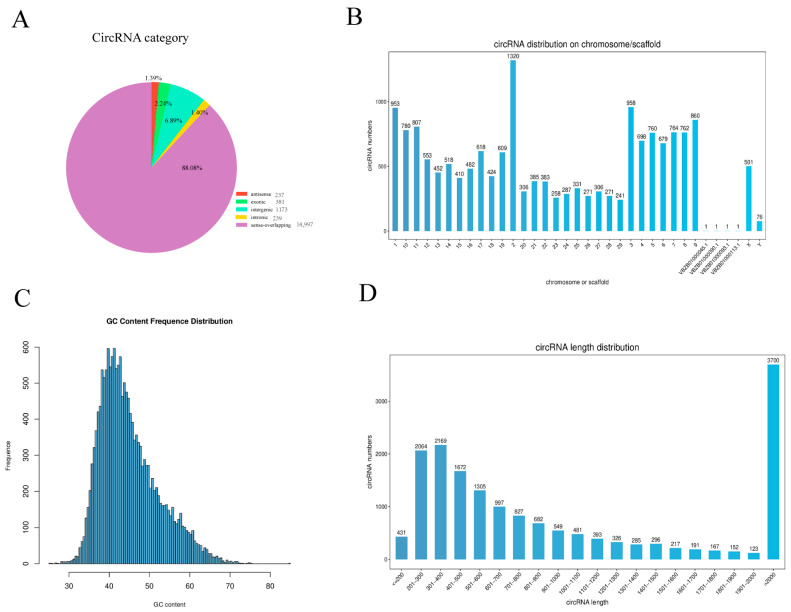
Expression profile analysis of CircRNAs. (**A**) Classification of CircRNA in the longissimus dorsi muscle. (**B**) Distribution of circular RNA quantities across chromosomes. (**C**) GC content distribution of CircRNA. (**D**) Sequence length distribution of CircRNA.

These purified data fully satisfy the quantitative and quality control requirements for subsequent circular RNA analysis. Following screening and coding capacity assessment, this study identified a total of 17,027 CircRNAs, with a systematic analysis conducted on their gene origins. The newly discovered CircRNAs were categorized into five classes: antisense (*n* = 237), exonic (*n* = 381), intergenic (*n* = 1173), intronic (*n* = 239), and sense-overlapping (*n* = 14,997) CircRNAs. Sense-overlapping CircRNAs constituted the largest proportion (88%), followed by intergenic (7%), exonic (2%), antisense (1%), and intronic (1%) types (Figure 1A). Chromosomal distribution data revealed near-universal presence across all chromosomes, with the highest enrichment on chromosome 2, followed by chromosomes 1 and 3 (Figure 1B). The GC content of circular RNA sequences ranged from 46.73% to 51.62% (Figure 1C), meeting fundamental compositional requirements. Regarding the length distribution, CircRNAs exceeding 2000 bp were the most numerous (3700), followed by those between 301 and 400 bp (2169) (Figure 1D).

### 3.3. Identification and Expression Analysis of DE CircRNAs

**Figure 2:**
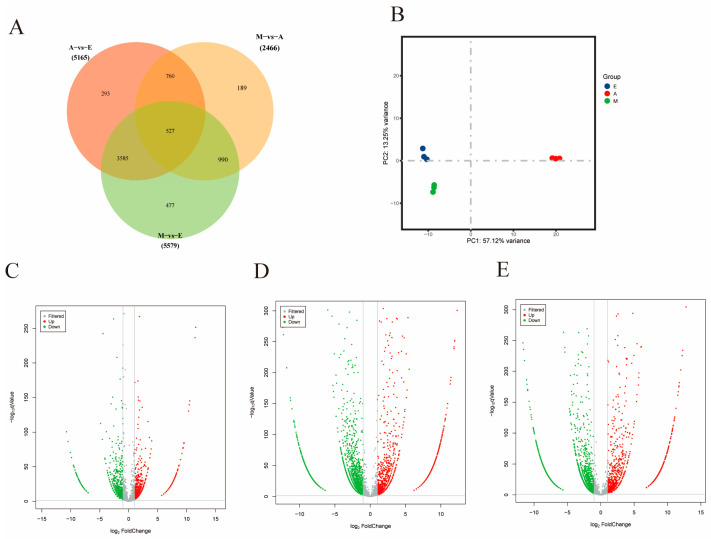
DE CircRNAs in skeletal muscle of Datong yak at different growth stages. (**A**) Distribution of DE CircRNAs revealed by Venn diagram. (**B**) Principal component analysis of nine samples. (**C**) Volcano plot of DE CircRNAs (M vs. A). (**D**) Volcano plot of DE CircRNAs (M vs. E). (**E**) Volcano plot of DE CircRNAs (A vs. E).

We screened for DE CircRNAs in skeletal muscle tissue from yak calves across different age groups using a *p*-value < 0.05 and |log2FC| ≥ 1 as selection criteria, identifying a total of 6821 differentially expressed CircRNA (Figure 2A). Principal component analysis (PCA) was employed to evaluate the expression patterns of these CircRNAs, revealing that biological samples from each growth stage clustered together (Figure 2B). Compared to Group A, Group M exhibited 1543 upregulated and 923 downregulated DE CircRNAs (Figure 2C), while Group E showed 1420 upregulations and 3745 downregulations (Figure 2E). Relative to Group E, Group M demonstrated 1965 upregulations and 3614 downregulations (Figure 2D). These findings lay the groundwork for the subsequent screening of CircRNAs associated with yak skeletal muscle development.

### 3.4. GO Function Enrichment Analysis of Target Genes

To elucidate the regulatory roles of differentially expressed CircRNAs (DE CircRNAs) in the longissimus dorsi muscle of Datong yaks, GO functional annotation analysis was conducted.

**Figure 3:**
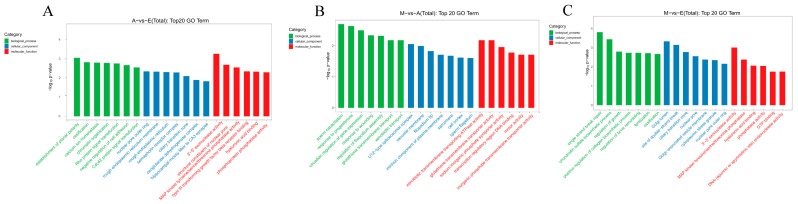
GO enrichment analysis of DE CircRNA target genes. (**A**) A vs. E. (**B**) M vs. A. (**C**) M vs. E.

GO analysis revealed significant enrichment in 175, 108, and 146 GO terms when comparing Group A versus Group E, Group M versus Group A, and Group M versus Group E respectively. Terms significantly enriched and associated with skeletal muscle development included establishment of planar polarity (GO:0001736), ossification (GO:0001503), calcium ion homeostasis (GO:0055074), Rho protein signal transduction (GO:0007266), Cdc42 protein signal transduction (GO:0032488), sarcomere (GO:0030017), cell cortex (GO:0005938), regulation of bone remodeling (GO:0046850), regulation of growth (GO:0040008), positive regulation of collagen biosynthetic process (GO:0032967), MAP kinase tyrosine/serine/threonine phosphatase activity (GO:0017017), and GTP binding (GO:0005525) pathways (Figure 3).

### 3.5. KEGG Function Enrichment Analysis of Target Genes

**Figure 4:**
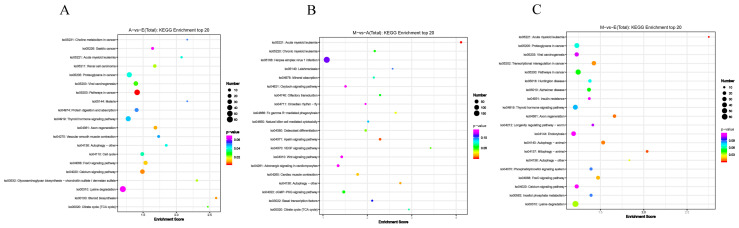
KEGG enrichment analysis of DE CircRNA target genes. (**A**) A vs. E. (**B**) M vs. A. (**C**) M vs. E.

The KEGG database analysis of transcriptomic results revealed that 18, 7, and 10 KEGG pathways were significantly enriched in comparisons between Group A and Group E, Group M and Group A, and Group M and Group E, respectively. Significantly enriched pathways included the calcium signaling pathway (ko04020), FoxO signaling pathway (ko04068), cell cycle (ko04110), thyroid hormone signaling pathway (ko04919), and mitophagy—animal (ko04137) (Figure 4).

### 3.6. Screening of DE CircRNAs Associated with Skeletal Muscle Development

We screened and selected the top 20 DE CircRNAs linked to skeletal muscle development (Table 2).

### 3.7. Verification of RNA-Seq Results

**Figure 5:**
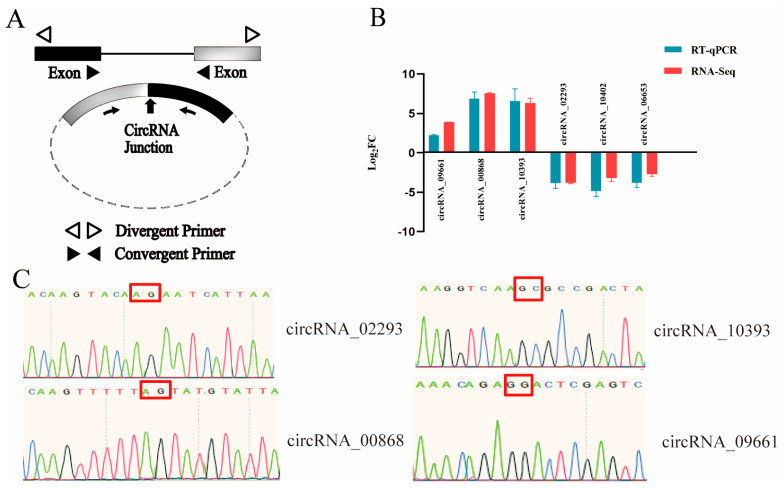
Validation of DE CircRNAs. (**A**) Schematic diagram of primer design for qRT-PCR detection of differentially expressed CircRNAs. (**B**) Comparative analysis of six DE CircRNA expression levels obtained by RNA-seq and qRT-PCR, upregulated CircRNAs (CircRNA_09661, CircRNA_00868, CircRNA_10393) and downregulated CircRNAs (CircRNA_02293, CircRNA_10402, CircRNA_06653). (**C**) Sanger sequencing results for the four differentially expressed CircRNAs. β-actin was used as the housekeeping gene. qRT-PCR was performed with three replicates. Data are shown as the mean ± standard error of the mean (SE), with error bars representing the SE. The circularization site sequences of CircRNAs in the circbase database are as follows: CircRNA_02293 (ACAAGTACAAGAATCATTAA); CircRNA_10393 (AAGGTCAAGCGCCGACTA); CircRNA_00868 (CAAGTTTTTAGTATGTATTA); CircRNA_09661 (AAACAGAGGACTCGAGTC).

To further confirm the reliability of the RNA-seq data, six CircRNAs were randomly selected and subjected to qRT-PCR validation. The results demonstrated a high degree of concordance between the qRT-PCR and RNA-seq data in terms of expression trends (Figure 5B). Among these, four CircRNAs (CircRNA_09661, CircRNA_00868, CircRNA_10393, and CircRNA_02293) were further confirmed by Sanger sequencing (Figure 5C). Taken together, these experimental findings provide strong evidence supporting the authenticity of the identified CircRNAs and further substantiate the robustness and reliability of the RNA-seq dataset.

### 3.8. Analysis of CircRNA–miRNA–mRNA Regulatory Networks

**Figure 6:**
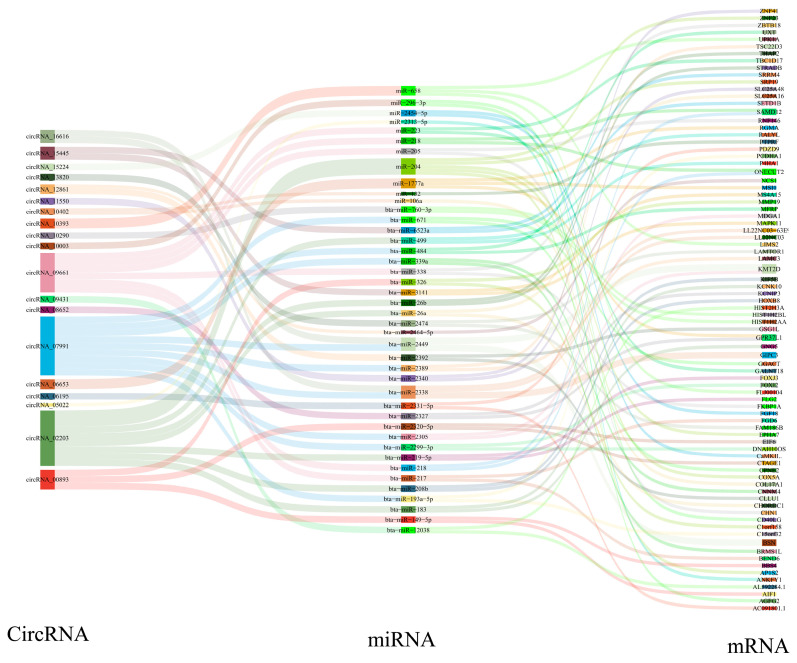
The CircRNA–miRNA–mRNA regulatory network. Sankey diagram of the ceRNA network in the yak’s longissimus dorsi muscle.

To assess the miRNA sponge potential of the identified CircRNAs, we predicted CircRNA–miRNA interactions using miRanda (v3.3a) and TargetScan (v7.0). Leveraging 20 DE CircRNAs, 42 target miRNAs, and 84 associated mRNAs, we constructed 89 CircRNA–miRNA–mRNA regulatory networks implicated in skeletal muscle development (Figure 6, Table S1). The analysis further indicated the cooperative regulation of identical miRNAs by multiple CircRNAs, exemplified by CircRNA_07991 and CircRNA_16616, both targeting bta-miR-2338.

## 4. Discussion

Muscle development represents a complex biological process precisely regulated by a multi-level molecular network, involving myoblast proliferation, differentiation, and fusion, as well as myofibril maturation and metabolic conversion [7]. Recently, non-coding RNAs have attracted growing attention in post-transcriptional regulation [13]. However, CircRNA functions and regulatory networks during yak skeletal muscle development at different stages have not been fully explored. Based on this, this study conducted RNA sequencing analysis on the latissimus dorsi muscle tissues of Datong yak during the fetal stage (90 days of gestation), juvenile stage (6 months), and adult stage (3 years). It systematically identified the CircRNA expression profiles at each developmental stage and constructed a competitive endogenous RNA regulatory network linked to CircRNA. The aim was to provide a new theoretical foundation for elucidating the molecular mechanisms underlying yak muscle development and to offer scientific evidence for genetic improvement.

By comparing three developmental stages, we identified a total of 6821 differentially expressed CircRNAs. To gain insight into the potential biological functions of differentially expressed CircRNAs, GO functional annotation and KEGG pathway enrichment analyses were systematically conducted to elucidate their associated biological processes and signaling pathways. GO analysis revealed that differentially expressed CircRNAs were significantly enriched in “muscle sarcomere” and “calcium ion homeostasis”. Sarcomeres serve as the fundamental units of skeletal muscle contraction, and their proper assembly is crucial for muscle fiber function [23]. The “calcium ion homeostasis” process directly governs muscle excitation–contraction coupling. Precise regulation of intracellular calcium ion concentration is a key factor in triggering muscle contraction and influencing muscle tenderness [24]. The enrichment results suggest that differentially expressed CircRNAs may be involved in the regulation of muscle fiber structure and physiological properties. Additionally, “Rho protein signal transduction” and “Cdc42 protein signaling” pathways were also notably enriched. The Rho GTPase family (including RhoA, Rac1, Cdc42) comprises key molecules regulating cytoskeletal reorganization, cell migration, and morphogenesis, playing vital roles in myoblast fusion and tubule formation [25]. This finding suggests that CircRNA may influence muscle development by regulating cytoskeletal dynamics during myogenesis. KEGG pathway enrichment analysis further showcased that notably enriched pathways included “FoxO signaling”, “calcium signaling”, “thyroid hormone signaling”, and “cell cycle”. FoxO transcription factors are key regulators of autophagy, protein degradation, and metabolism. In skeletal muscle, FoxO pathway activity is closely associated with muscle atrophy, yet it also participates in regulating the differentiation balance of myoblasts during development [26]. The calcium signaling pathway is not only central to muscle contraction; its members, such as calmodulin and calcium/calmodulin-dependent protein kinase (CaMK), also regulate muscle gene expression and metabolism [27]. Thyroid hormones are crucial regulators of animal growth and development, promoting protein synthesis and myofibrillar type conversion [28]. The enrichment of this pathway suggests that the host genes of these CircRNAs may regulate myoblast proliferation activity, thereby influencing the muscle fiber number (hyperplasia). Additionally, the enrichment of the “autophagy–animal” pathway warrants attention, as selective mitochondrial autophagy (mitophagy) is crucial for maintaining muscle mitochondrial quality and energy metabolic homeostasis [29]. In conclusion, these pathway enrichment results provide mechanistic insights into how CircRNAs coordinate multiple signaling cascades to regulate skeletal muscle development in yaks.

From the CircRNAs exhibiting the most significant differential expression, we selected the top 20 CircRNAs linked to skeletal muscle development genes for in-depth analysis. The host genes of these CircRNAs span multiple levels including transcriptional regulation, signal transduction, cytoskeletal organization, and metabolism.

In terms of transcriptional regulation and signal transduction, CircRNA_15445 originates from MEF2C, a core transcription factor that regulates muscle-specific gene expression and terminal differentiation, synergizing with MRFs to activate genes encoding muscle structural and functional proteins [30]. Its circular form may fine-tune muscle differentiation programs by binding miRNAs to regulate MEF2C mRNA stability or translation efficiency. CircRNA_02203 originates from TCF12, an E-protein family member that heterodimerizes with MyoD to enhance DNA-binding capacity, serving as an essential myogenic co-factor [31]. CircRNA_15224 originates from FGFR4. The FGF signaling pathway regulates myoblast proliferation and satellite cell activity. In pigs, FGFR4 regulates the Wnt/β-catenin pathway by binding miR-107, influencing myoblast proliferation [32]. Similar mechanisms may exist in yaks, though validation is needed.

Regarding cytoskeletal structure and sarcomere integrity, CircRNA_10393 originates from LDB3 (ZASP), a critical Z-disc component maintaining myotonic integrity and signal transduction. CircRNA_09661 (SORBS1) is involved in cell adhesion, cytoskeletal organization, and insulin signaling [33,34]. In cattle, SORBS1 variants are associated with meat tenderness and marbling [35]; its CircRNA form in yaks may similarly influence meat quality traits.

With respect to metabolism, energy homeostasis, and autophagy, CircRNA_13820 originates from the PPARGC1A gene (PGC-1α) and serves as an important regulator of mitochondrial biogenesis and oxidative metabolism, influencing the composition of muscle fiber types [36]. CircRNA_10003 is derived from the EEF2K gene, which inhibits protein translation by phosphorylating eukaryotic elongation factor 2 (EEF2) and acts as a critical regulator of translation rates under energy stress conditions (e.g., hypoxia) [37]. As a species adapted to high-altitude environments, yak muscle cells may utilize such CircRNAs to regulate energy metabolism under hypoxic conditions. CircRNA_07991 (TK2) plays a pivotal role in mitochondrial DNA synthesis, and its dysfunction can lead to mitochondrial myopathy [38]. The differential expression patterns (upregulation or downregulation) of these key CircRNAs at different developmental stages strongly suggest their stage-specific involvement in regulating multiple biological processes of yak skeletal muscle, including proliferation, differentiation, structural assembly, and metabolic adaptation.

## 5. Limitations and Future Perspectives

This study has several limitations. First, only three biological replicates were used per developmental stage. Although this meets the minimum acceptable standard for RNA-seq differential expression analysis, and edgeR has been validated to control false discovery rates with as few as three replicates per group [20], the limited sample size reduces the statistical power for detecting CircRNAs with minor expression changes and may increase the false discovery rates. To mitigate this, we applied a stringent screening criterion (|log2FC| ≥ 1) and validated the key findings via qRT-PCR. Nevertheless, future studies with larger sample sizes (n ≥ 6 per group) are needed to confirm and expand these findings. Second, the constructed ceRNA network is entirely based on bioinformatic predictions (miRanda, TargetScan, and miRTarBase), which are prone to false positives; therefore, the predicted CircRNA–miRNA–mRNA axes should be regarded as hypotheses requiring experimental validation (e.g., dual luciferase reporter assays, RNA immunoprecipitation) rather than established mechanisms. Third, this study did not integrate meat quality traits (e.g., tenderness, intramuscular fat content, shear force, color) or muscle morphological parameters (e.g., fiber type, diameter, density) from different developmental stages, nor did it directly correlate the identified ceRNA networks with yak meat quality characteristics. Consequently, the specific regulatory roles of core axes on meat quality traits remain unclear, limiting the direct applicability of these findings to molecular breeding for meat quality improvement. Based on these limitations, future research should prioritize experimental validation of the predicted ceRNA axes (e.g., CircRNA_15445–miRNA–MEF2C) using yak primary myoblasts, integrate meat quality phenotypes and muscle histological data to directly link specific ceRNA networks with economically important traits, and conduct comparative studies across different yak populations or between yaks and cattle to reveal species-specific regulatory mechanisms underlying skeletal muscle development and meat quality formation.

## 6. Conclusions

In this study, we systematically characterized the genome-wide expression landscape of CircRNAs in the longissimus dorsi muscle of Datong yaks across three critical developmental stages: fetal (90 days), juvenile (6 months), and adult (3 years). A large number of CircRNAs were identified, exhibiting stage-specific expression patterns and significant enrichment in biological processes closely related to skeletal muscle development, including sarcomere assembly, calcium ion homeostasis, and the FoxO signaling pathway.

More importantly, this research revealed the dynamic regulatory network characteristics of CircRNAs during yak skeletal muscle development. The core CircRNAs screened in this study, including CircRNA_15445 (originating from MEF2C) and CircRNA_15224 (originating from FGFR4), along with their ceRNA regulatory networks, are involved in critical muscle development processes such as myocyte differentiation, muscle fiber assembly, and cellular signal transduction. These processes constitute the essential molecular basis for the formation of yak meat quality characteristics. For instance, MEF2C-regulated muscle fiber differentiation directly influences muscle fiber type composition, thereby determining meat tenderness and flavor. These core CircRNA–ceRNA regulatory axes identify key candidate targets for investigating the molecular mechanisms governing yak meat quality formation. Nevertheless, this study has several limitations, including the relatively small sample size (n = 3 per group) and the reliance on bioinformatic predictions for ceRNA interactions, which require experimental validation (e.g., dual luciferase reporter assays, RNA immunoprecipitation).

In conclusion, our findings provide a novel theoretical foundation for understanding the epigenetic regulation of yak skeletal muscle development and offer promising candidate CircRNA targets for future molecular breeding strategies aimed at improving yak meat quality.

## Figures and Tables

**Table 1:** RNA-seq data overview.

Sample	Raw Reads	Clean Reads	Q30 (%)	Total Mapped	Multiple Mapped	Unique Mapped
A1	108.30 M	103.28 M	94.37%	97,318,368 (94.23%)	14,636,057 (14.17%)	82,682,311 (80.06%)
A2	104.59 M	99.70 M	94.39%	94,704,930 (94.99%)	13,668,177 (13.71%)	81,036,753 (81.28%)
A3	101.43 M	96.45 M	94.03%	90,343,986 (93.67%)	12,720,717 (13.19%)	77,623,269 (80.48%)
M1	95.00 M	94.35 M	96.78%	88,447,158 (93.10%)	13,845,837 (14.57%)	74,601,321 (78.52%)
M2	95.04 M	92.10 M	96.87%	87,745,255 (92.32%)	13,258,787 (13.95%)	74,486,468 (78.37%)
M3	96.06 M	95.43 M	96.69%	89,625,215 (93.30%)	15,233,748 (15.86%)	74,391,467 (77.44%)
E1	110.56 M	105.51 M	94.24%	99,289,344 (94.11%)	9,599,303 (9.10%)	89,690,041 (85.01%)
E2	112.59 M	107.85 M	94.66%	101,577,932 (94.19%)	9,906,302 (9.19%)	91,671,630 (85.00%)
E3	117.51 M	111.70 M	93.94%	104,774,361 (93.80%)	11,046,983 (9.89%)	93,727,381 (83.91%)

**Table 2:** Twenty key DE CircRNAs linked to skeletal muscle development.

CircRNAs ID	Source Gene	*p*-Value	Up/ Down	CircRNA Size
CircRNA_15445	MEF2C	5.62 × 10^−4^	Up	373
CircRNA_10402	MAPK8	4.47 × 10^−4^	Down	665
CircRNA_06653	CDC42	1.94 × 10^−4^	Down	228
CircRNA_10003	EEF2K	3.46 × 10^−4^	Up	436
CircRNA_09661	SORBS1	3.28 × 10^−4^	Up	412
CircRNA_05022	RGMA	2.60 × 10^−3^	Up	1935
CircRNA_02203	TCF12	4.25 × 10^−4^	Down	1029
CircRNA_15224	FGFR4	3.28 × 10^−4^	Up	963
CircRNA_10393	LDB3	1.96 × 10^−4^	Up	1944
CircRNA_10290	MYPN	3.50 × 10^−4^	Down	1068
CircRNA_13820	PPARGC1A	3.38 × 10^−4^	Up	1142
CircRNA_00893	DYPK1A	1.08 × 10^−3^	Up	283
CircRNA_11550	DOCK7	3.49 × 10^−4^	Down	1538
CircRNA_07991	TK2	3.37 × 10^−4^	Up	257
CircRNA_09431	DST	1.97 × 10^−3^	Down	2013
CircRNA_09513	MCUR1	3.06 × 10^−3^	Up	224
CircRNA_16616	AMOT	4.35 × 10^−4^	Up	499
CircRNA_08652	ATG7	9.44 × 10^−4^	Down	228
CircRNA_12861	MYBPC1	2.41 × 10^−4^	Down	453
CircRNA_06195	STAT5B	4.11 × 10^−4^	Down	1477

## Data Availability

Data are available upon request due to privacy/ethical restrictions.

## Supplementary Materials

The following supporting information can be downloaded at https://www.mdpi.com/article/10.3390/ani16101497/s1, Table S1. Information on qRT-PCR primer sequences. Table S2. The DE CircRNA–miRNA–mRNA interaction network.
